# Polymorphisms and expressions of ADSL, MC4R and CAPN1 genes and their effects on economic traits in Egyptian chicken breeds

**DOI:** 10.1007/s11033-023-08999-w

**Published:** 2023-12-10

**Authors:** Dalia M. Aboelhassan, Hassan R. Darwish, Hayam Mansour, Hesham Abozaid, Inas S. Ghaly, Hasnaa A. Radwan, Eman R. Hassan, Ibrahim M. Farag

**Affiliations:** 1 Department of Cell Biology, Biotechnology Research Institute, National Research Centre, 33st El Bohouth, Dokki, Giza, 12622 Egypt; 2 Department of Animal Production, Agricultural and Biology Institute, National Research Centre, Giza, 12622 Egypt; 3 Department of Poultry Disease, Veterinary Research Institute, National Research Centre, Giza, 12622 Egypt

**Keywords:** PCR-SSCP, Real-time PCR, ADSL, MC4R and CAPN1 genes, Economic traits, Chicken breeds

## Abstract

In recent years, strategic plans for poultry production have emphasized quantitative traits, particularly body weight and carcass traits (meat yield), in response to overpopulation challenges. Candidate genes such as adenylosuccinate lyase (ADSL), melanocortin-4-receptor (MC4R), and calpain 1 (CAPN1) have played vital roles in this context due to their associations with muscle growth and body composition. This study aims to investigate the influence of polymorphisms and gene expressions of the aforementioned genes on body weight (BW), growth rate (GR), breast weight (BrW), and thigh weight (TW) across four distinct chicken breeds: Fayoumi, Matrouh, Mamourah, and Leghorn. The use of PCR-SSCP analysis revealed genetic polymorphisms through the identification of various patterns (genotypes) within the three examined genes. The ADSL, MC4R, and CAPN1 genes exhibited five, three, and two different genotypes, respectively. These polymorphisms displayed promising connections with enhancing economically significant production traits, particularly BW, BrW and TW. Furthermore, gene expression analyses were conducted on breast and thigh tissues obtained from the chicken breeds at 60 days of age, where ADSL and MC4R exhibited a noteworthy up-regulation in Fayoumi and Matrouh breeds, and down-regulation in Mamourah and Leghorn. In contrast, CAPN1 expression decreased across most breeds with a slight increase noted in Fayoumi breed. In conclusion, this investigation underscores the substantial impact of ADSL, MC4R, and CAPN1 genes on economically important production traits within Egyptian domestic chicken breeds. Consequently, these genes emerge as significant molecular markers, holding potential utility in avian selection and breeding programs aimed at enhancing productive performance.

## Introduction

Recently, the focus of poultry selection strategies has predominantly centered around quantitative traits aimed at enhancing growth rates and meat yield, driven by the imperative to meet the escalating demands of a burgeoning global population [1–3]. This heightened emphasis on such traits has become imperative to address the impending challenge of accommodating the projected nine-billion-strong human population by 2050, which is anticipated to necessitate a staggering 70 to 100% increase in food production relative to previous Figs. [2, 4–6]. Consequently, the pursuit of enhanced growth rates and refined body composition traits, particularly those pertaining to breast and thigh weights, has emerged as a pivotal objective in various research pursuits. This collective effort serves to minimize costs, optimize final products, and effectively cater to the mounting global populace [2, 5, 6]. Of paramount importance within the realm of chicken production are growth rates and body composition, notably the weights of breast and thigh muscles. These traits, tightly linked to quantitative trait loci (QTLs), have been underscored in previous studies [2, 5, 7–10]. Notably, the elucidation of QTLs chiefly hinges upon the candidate gene approach [5, 11, 12], which has emerged as a potent means to discern genetic variations underpinning chicken traits of interest. This approach, underscored by its efficacy in identifying major alleles or genotypes associated with trait enhancements, has demonstrated its power in determining the genetic fabric of pivotal poultry attributes [2, 11, 13]. Corroborative studies have repeatedly validated the nexus between candidate gene polymorphisms (specifically SNPs or genotypes) and a spectrum of chicken production traits encompassing growth and body composition [2, 3, 14–17]. Furthermore, gene expression measurements have emerged as a reliable and informative conduit for identifying chicken genes germane to economically pivotal traits, thereby furnishing insights for optimal gene expression levels conducive to superior poultry production quantities and qualities [2, 6, 9]. This methodology, which allows for early prognosis and inter-breed comparisons, aids in steering the trajectory of chicken breeding strategies towards more productive outcomes [2, 6]. Prominently situated within the pantheon of candidate genes orchestrating quantitative production traits in chickens are melanocortin-4-receptor (MC4R), adenylosuccinate lyase (ADSL), and calpain 1 (CAPN1) genes [2, 17, 18]. The MC4R gene occupies a pivotal role in feeding regulation and energy homeostasis [19], while concurrently exerting its influence over physiological processes spanning lipolysis, immunology, and adrenal steroidogenesis [18]. Studies have elucidated the significant interplay between MC4R gene activity and body weight in chickens, underscoring distinct MC4R genotypes as determinants of protein functions integral to body weight dynamics [2, 13, 20, 21]. Similarly, the CAPN1 gene emerges as a critical actor in postmortem meat tenderness and muscle growth regulation in chickens [17, 22]. Its role in the degradation of myofibrillar proteins and myosin underscores its profound implications for muscle biology [23]. A tapestry of research endeavors has substantiated the significant association between CAPN1 gene function and body weight attributes in avian subjects [2, 17, 23]. The ADSL gene, pivotal for inosine 5’-monophosphate (IMP) content and essential for maintaining normal cell division and metabolism [24, 25], is intrinsically woven into the intricate fabric of chicken body weight modulation. The IMP compound, intricately linked to purine biosynthesis, reverberates with significance for chicken body weight dynamics, accentuating the gene’s role in shaping these pivotal production traits [2, 26–28]. Against this backdrop, the present study embarks on an exploration of the polymorphisms and expressions of MC4R, ADSL, and CAPN1 genes, anchoring its investigation in the domain of growth and body composition traits within distinct chicken breeds indigenous to Egypt. The breeds encompass Fayoumi, Matrouh, Mamourah, and Leghorn varieties, collectively endeavoring to unravel the intricate genetic tapestry that shapes these birds’ productivity and qualities.

## Materials and methods

### Chicken populations

This study followed institutional, national, and international guidelines for the ethical treatment and use of animals. The ethical protocols and animal care procedures complied with the Medical Research Ethical Committee (MREC) and Animal Care and Use Committee (ACUC) of the National Research Centre. Four distinct chicken populations, comprising Mamourah (a hybrid strain combining Alexandria and Dokki-4 strains), Matrouh (a hybrid strain originating from Dokki-4 and Leghorn strains), Leghorn (an imported strain), and Fayoumi (a native Egyptian strain), constituted the subjects of this study. All chickens were raised under identical environmental and management conditions. Experimental diets adhered to the nutritional requirements specified by the NRC (National Research Council). These diets consisted of yellow maize, soybean cake, full-fat extruded soybean, guar korma cake, dicalcium phosphate, mixed vitamins, and minerals, hydroxymethionine, analog calcium, sodium bicarbonate, calcium carbonate, and table salt. Nutritional analyses revealed a crude protein content of not less than 23%, crude fat of not less than 5.76%, a crude fiber content of not exceeding 4.2%, and a representative energy value of no less than 3000 Kilocalories per kilogram of diet following the guidelines of animal care unit of National Research Centre. Growth Rate and Sample Collection: Live body weights were assessed at both one and 60 days of age to ascertain the growth rate (final weight - initial weight/initial weight). Blood samples were collected from the brachial veins of 80 broilers using standard venipuncture techniques. These samples were promptly transferred to blood collection tubes containing heparin as an anticoagulant factor, after which they were preserved at -20 °C until DNA isolation procedures were undertaken. Upon reaching 60 days of age, the chickens were humanely slaughtered, and the subsequent evisceration and dissection procedures were conducted. This facilitated the determination of breast and thigh weights. Within the context of this investigation, 20 breast samples and 10 thigh samples. from each breed were utilized.

#### Tissue Preparation and Gene Expression Analysis

Tissues, encompassing breast and thigh specimens were swiftly excised and snap-frozen in liquid nitrogen. These samples were subsequently stored at -80 °C, ensuring their preservation for subsequent gene expression analyses.

### **Genetic polymorphism analysis**

#### DNA extraction

Genomic DNA was extracted from whole blood samples utilizing the salting-out method, with some modifications [29]. The purity and relative integrity of the obtained genomic DNA were assessed through 1.5% agarose gel electrophoresis. Additionally, DNA concentration was determined using a NanoDrop 2000 Spectrophotometer (Thermo Fisher Scientific, Waltham, MA, USA), with final DNA concentrations ranging from 2 to 10 ng/µL.

#### PCR amplification

For polymerase chain reaction (PCR), specific primer pairs targeting MC4R, CAPN1, and ADSL genes were employed in Table 1. PCR amplification reactions were performed using DreamTaq Green PCR Master Mix (Thermo Fisher Scientific, Waltham, MA). Each 25-µL reaction mixture comprised 12.5 µL of DreamTaq Green PCR Master Mix (2×), 0.5 µL each of forward and reverse primers (10 µM), 1.5 µL of template DNA, and 10 µL of nuclease-free water. The PCR conditions for MC4R and CAPN1 involved an initial denaturation at 94 °C for 6 min, followed by 35 cycles of 94 °C for 45 s, 58 °C for 45 s, and 72 °C for 1 min, with a final extension step at 72 °C for 9 min. For ADSL, the PCR conditions comprised an initial denaturation at 95 °C for 6 min, followed by 35 cycles of 95 °C for 40 s, 56 °C for 35 s, and 72 °C for 50 s, with a final extension at 72 °C for 9 min.

**Table 1:** List of primer sequences used for genotyping by PCR-SSCP and gene expression analysis using real-time PCR

Primer name	Primer sequence	Product size
ADSL_SNP	F: CTTTCTCCTCCGCAGTCAC R: AGCACCTTCGTCTTCGTTTT	230 bp
MC4R_SNP	F: TTCGCCCATGTACTTC R: CTGGAGGGCATAAAAGATAGT	241 bp
CAPN1_SNP	F: AGGGGTAGGGTAATAGAACTA R: ACCGCCAGCCATCAAAT	233 bp
ADSL_Expr	F: CGAGAGGAAGAAGTTCGGCA R: TGATGGGAAGCCCAAGTGAC	80 bp
MC4R_Expr	F: GTCAAGCGTGTAGGGGTCAT R: CTTTCATGTTGGCCCCTTGG	241 bp
CAPN1_Expr	F: CGAGGGCGAAATCGATGAGA R: CATAGGCGCTCATACTGCCA	299 bp
GAPDH-Expr	F: GTCAAGGCTGAGAACGGGA R: GCCCATTTGATGTTGCTGG	86 bp

*

#### PCR-SSCP analysis

To determine the genotypes of MC4R, CAPN1, and ADSL in various chicken breeds, PCR single-strand conformation polymorphism (PCR-SSCP) was employed, following the methodologies outlined by Zhang et al. [23], Wang et al. [21], and Wu et al. [26], respectively. Briefly, the amplified products were combined with a quarter volume of loading buffer (composed of 98% formamide, 0.09% xylene cyanole FF, and 0.09% bromophenol blue), heated for 10 min at 98 °C, and promptly cooled on ice. The mixture was loaded onto a 10% polyacrylamide gel and subjected to electrophoresis for 4 to 5 h at 140 V/cm. Subsequent to electrophoresis, individual SSCP banding patterns were ascertained under visible light.

### Gene expression analysis

#### RNA extraction and real-time PCR analysis

Total RNA extraction from breast and thigh tissues was executed using TRIzol Reagent (Invitrogen; Thermo Fisher Scientific). Extracted RNA was quantified utilizing a NanoDrop 2000 spectrophotometer (Thermo Fisher Scientific) and reversed transcribed using a High Capacity cDNA Reverse Transcription Kit (Applied Biosystems; Thermo Fisher Scientific), as per the manufacturer’s protocols. Real-time quantitative PCR (qPCR) reactions were carried out on a Light Cycler 480 System (Roche Diagnostics GmbH, Mannheim, Germany). For expression data normalization, Glyceraldehyde-3-phosphate dehydrogenase (GAPDH) served as the internal reference gene. Primer sequences for gene expression analysis were designed according to Kubota et al. [2] as shown in Table 1. Each qPCR reaction contained 20 µL volume, comprising 10 µL SYBR Green I Master (Roche Diagnostics GmbH), 1 µL of both forward and reverse primers, 2 µL cDNA template, and 6 µL nuclease-free water. The thermocycling program involved initial denaturation at 95 °C for 30 s, followed by 45 cycles of 95 °C for 5 s and 59 °C for 20 s for MC4R and ADSL, or 45 cycles of 95 °C for 5 s and 60 °C for 20 s for CAPN1. Triplicate amplification reactions were performed for each gene. Following qPCR, melting curve analysis was conducted to validate the amplification of specific PCR fragments. Gene expression levels, relative to GADPH, were evaluated using the 2^−ΔCt^ method [30].

### Statistical analysis

All statistical analyses were performed using SPSS software. Data were subjected to one-way analysis of variance (ANOVA), followed by Duncan’s post hoc test for intergroup comparisons [31]. Values are presented as mean ± SE, with statistical significance set at P < 0.05.

## Results

### PCR-SSCP analysis

**Fig. 1:**
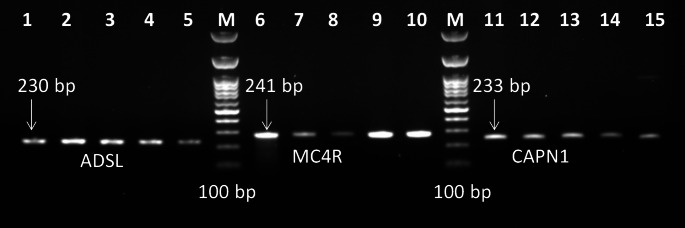
PCR amplification of three genes (ADSL, MC4R, and CAPN1), where L: 1–5 showed PCR amplification of ADSL gene at 230 bp, L: 6–10 showed PCR amplification of MC4R gene at 240 bp, and L: 11–15 showed PCR amplification of CAPN1 gene at 233 bp, L: M is 100 bp ladder

**Fig. 2:**
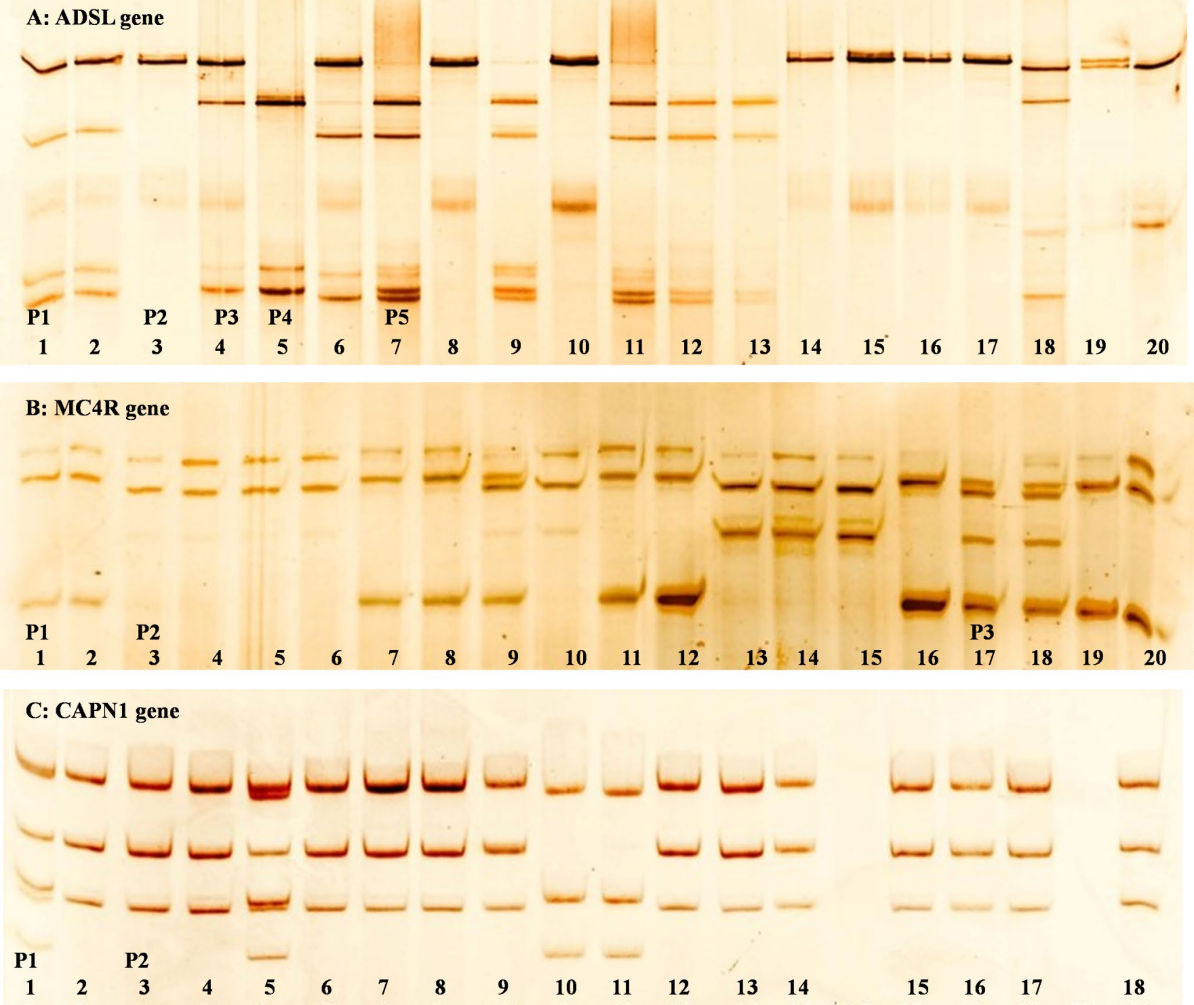
Detection of different patterns of three different genes (ADSL, MC4R, and CAPN1) on polyacrylamide gel in four chicken breeds (Fayomi, Matrouh, Mamourah, and Leghorn), where **A**: ADSL gene showed 5 different patterns, **B**: MC4R gene showed three different patterns and **C**: CAPN1 showed two different patterns

The PCR-SSCP procedure was effectively developed to screen individuals within the population. This study focused on amplifying, denaturing, and subjecting three target gene fragments to polyacrylamide gel electrophoresis to identify SSCP polymorphisms. The expected PCR product sizes were achieved (230 bp for ADSL, 241 bp for MC4R, and 233 bp for CAPN1 genes) (Fig. 1). Usage of PCR-SSCP analysis showed that the aforementioned genes had different patterns, each pattern possessed different bands (Fig. 2).

### Genetic polymorphisms in ADSL gene

PCR-SSCP results (Fig. 2A; Table 2) unveiled five patterns (P1, P2, P3, P4, and P5) across four chicken breeds: Fayoumi, Matrouh, Mamourah, and Leghorn. P1 and P2 were observed in all four breeds. However, P3, P4, and P5 were detected in three chicken breeds and were absent in one breed each; Matrouh, Mamourah, and Leghorn breeds, respectively.

**Table 2:** Frequency distribution of different patterns for ADSL, MC4R, and CAPN1 genes observed in four chicken breeds

		ADSL					MC4R			CAPN1	
Breed	No. of birds	F. of P1	F. of P2	F. of P3	F. of P4	F. of P5	F. of P1	F. of P2	F. of P3	F. of P1	F. of P2
**Fayoumi**	10	0.6	0.1	0.1	0.1	0.1	0.0	0.2	0.8	1.0	0.0
**Matrouh**	10	0.2	0.7	0.0	0.1	0.0	0.3	0.3	0.4	0.8	0.2
**Mamourah**	10	0.1	0.5	0.3	0.0	0.1	0.0	0.0	1.0	0.9	0.1
**Leghorn**	10	0.1	0.3	0.4	0.1	0.1	0.4	0.5	0.1	0.7	0.3

**F.: Frequency of the pattern. P1: Pattern 1. P2: Pattern 2. P3: Pattern 3. P4: Pattern 4. P5: Pattern 5.

### Association between PCR-SSCP patterns of ADSL gene and production traits in four chicken breeds

The impact of P1, P2, P3, P4, and P5 on economic traits: live body weight at 60 days of age (FW), growth rate (GR), carcass weight (CW), breast weight (BrW), and thigh weight (TW) is detailed in Table 3. Birds possessing P1 exhibited significant increases in FW, GR, CW, BrW, and TW, with Matrouh and Fayoumi breeds leading in these traits. The presence of P2 in Fayoumi breed significantly enhanced all the aforementioned economic traits compared to the other three breeds. For P3, Fayoumi breed displayed significant improvements in FW, GR, and CW compared to Mamourah and Leghorn breeds. Birds harboring P3 in Mamourah showed significant increases in BrW and TW compared to Fayoumi and Leghorn breeds. In terms of P4, Fayoumi breed showed significant improvements in FW, GR, CW, and slight enhancement in BrW compared to the other two breeds. Leghorn chickens with P4 had a slight increase in TW compared to Fayoumi and Matrouh. Regarding P5, Fayoumi breed displayed significant improvements in FW, GR, and CW compared to Mamourah and Leghorn. Fayoumi also exhibited significant and minor increases in TW compared to Mamourah and Leghorn, respectively. Meanwhile, Mamourah birds with P5 showed significant BrW improvements compared to Fayoumi and Leghorn.

**Table 3:** Effect of SSCP patterns of ADSL gene on some economic production traits in four chicken breeds

Pattern (P)	Economic traits						
	Breeds	IW	FW	GR	CW	BrW	TW
P1	Fayoumi	30.8 ± 1.0^a^	723.5 ± 18.9^b^	22.6 ± 1.2^b^	694.3 ± 16.9^b^	236.3 ± 17.0^a^	267.8 ± 12.0^a^
	Matrouh	30.5 ± 0.5^a^	733.0 ± 13.0^b^	23.0 ± 1.0^b^	712.5 ± 12.5^b^	316.0 ± 11.0^b^	338.5 ± 8.5^b^
	Mamourah	39.0 ± 1.2^b^	530.0 ± 8.7^a^	12.6 ± 0.6^a^	513.0 ± 13.3^a^	247.0 ± 6.0^a^	238.0 ± 11.5^a^
	Leghorn	33.0 ± 0.6^a^	493.0 ± 4.0^a^	13.9 ± 0.6^a^	470.0 ± 17.3^a^	220.0 ± 8.8^a^	265.9 ± 5.8^a^
P2	Fayoumi	30.0 ± 0.6^a^	801.0 ± 5.8^b^	25.7 ± 1.2^c^	773.0 ± 11.5^b^	343.0 ± 5.8^b^	380.0 ± 5.8^c^
	Matrouh	29.5 ± 0.3^a^	503.0 ± 6.2^a^	16.1 ± 0.2^b^	498.5 ± 19.0^a^	236.0 ± 3.7^a^	232.8 ± 7.0^a^
	Mamourah	38.6 ± 0.4^c^	543.6 ± 12.4^a^	13.1 ± 0.6^a^	518.8 ± 13.6^a^	232.4 ± 11.4^a^	275.0 ± 5.7^b^
	Leghorn	32.67 ± 0.7^b^	542.0 ± 4.3^a^	15.6 ± 1.1^b^	530.3 ± 18.6^a^	245.0 ± 20.2^a^	268.0 ± 19.6^b^
P3	Fayoumi	34.0 ± 1.2^b^	650.0 ± 11.5^c^	18.1 ± 0.6^b^	620.0 ± 5.8^c^	220.0 ± 5.8^b^	211.0 ± 6.4^a^
	Matrouh	N/A	N/A	N/A	N/A	N/A	N/A
	Mamourah	37.6 ± 0.3^c^	560.3 ± 6.7^b^	13.9 ± 0. 8^a^	539.6 ± 9.8^b^	260.0 ± 5.8^c^	263.3 ± 3.3^b^
	Leghorn	31.5 ± 0.5^a^	458.3 ± 18.9^a^	13.6 ± 0.4^a^	437.5 ± 15.3^a^	162.3 ± 8.6^a^	222.8 ± 4.8^a^
P4	Fayoumi	35.0 ± 0.6^b^	776.0 ± 5.8^b^	21.1 ± 0.6^b^	743.0 ± 5.6^b^	260.0 ± 5.8^b^	224.0 ± 5.7^a^
	Matrouh	30.0 ± 1.2^a^	485.0 ± 6^a^	15.2 ± 0.5^a^	471.0 ± 5.6^a^	227.0 ± 6.0^b^	214.0 ± 6.0^a^
	Mamourah	N/A	N/A	N/A	N/A	N/A	N/A
	Leghorn	30.0 ± 1.1^a^	500.0 ± 17.3^a^	15.7 ± 1.1^a^	480.0 ± 17^a^	234.0 ± 7.3^b^	240.0 ± 11.5^a^
P5	Fayoumi	34.0 ± 0.6^b^	685.0 ± 2.0^c^	19.1 ± 0.5^b^	654.0 ± 3.5^c^	213.0 ± 4^a^	260.0 ± 7.6^b^
	Matrouh	N/A	N/A	N/A	N/A	N/A	N/A
	Mamourah	37.6 ± 0.5^c^	613 ± 6.0^b^	15.6 ± 0. 9^a^	593 ± 5.8^b^	253.0 ± 5.8^b^	232 ± 6.0^a^
	Leghorn	30.0 ± 1.5^a^	485 ± 5.8^a^	15.2 ± 0.6^a^	463 ± 5.7^a^	215 ± 6.0^a^	253 ± 5.8^ab^

*The data had been expressed as means ± SE. The values with different letters (a, b, c) within a column represent significant statistical differences P < 0.05. IW: Initial body weight, at one day age. FW: Final body weight at 60 days age. GR: Growth rate. CW: carcass weights. BrW: Breast weight. TW: thigh weight

### Genetic polymorphisms in MC4R

PCR-SSCP results (Fig. 2B; Table 2) revealed the presence of three patterns; P1, P2, and P3. Pattern P1 was identified in Matrouh and Leghorn breeds, but absent in Fayoumi and Mamourah birds. P2 was present in Fayoumi, Matrouh, and Leghorn breeds, while absent in Mamourah breed. Pattern P3 was observed in all four chicken breeds. Frequencies of P1, P2, and P3 are provided in Table 2.

### Association between PCR-SSCP patterns of MC4R gene and production traits in four chicken breeds

The impact of patterns P1, P2, and P3 on economic traits such as FW at 60 days of age, GR, CW, BrW, and TW were analyzed (Table 4). For chickens carrying P1, Leghorn breed exhibited an insignificant increase in FW compared to Matrouh breed. Conversely, Matrouh birds with P1 showed significant improvements in GR, CW, BrW, and TW compared to Leghorn breed. Regarding P2, Fayoumi chickens with this pattern displayed significant enhancements in FW, GR, and CW compared to other breeds, while BrW and TW values were significantly higher in Matrouh birds. As for P3, Fayoumi birds carrying this pattern exhibited significant improvements in FW and GR compared to other breeds. However, CW value was significantly higher in Mamourah breed than in Fayoumi and Matrouh breeds, with an insignificant increase compared to CW in Leghorn chickens. While no significant differences were observed in the BRW trait between the four breeds, Matrouh birds demonstrated the highest values. Additionally, Fayoumi breed, followed by Mamourah breed, exhibited a significant increase in TW compared to Matrouh breed and an insignificant increase compared to Leghorn birds.

**Table 4:** Effect of SSCP patters of MC4R gene on some economic production traits in four chicken breeds

Pattern (P)	Economic traits						
	Breeds	IW	FW	GR	CW	BrW	TW
P1	Fayoumi	N/A	N/A	N/A	N/A	N/A	N/A
	Matrouh	30.0 ± 0.6^a^	659.7 ± 21.0^b^	20.5 ± 1.0^b^	641.0 ± 21.9^b^	294.0 ± 23.5^b^	299.0 ± 10.6^b^
	Mamourah	N/A	N/A	N/A	N/A	N/A	N/A
	Leghorn	31.7 ± 0.6^a^	482.8 ± 12.9^a^	14.1 ± 0.7^a^	455.7 ± 19.1^a^	200.5 ± 20.3^a^	219.5 ± 8.9^a^
P2	Fayoumi	31.0 ± 1.0^a^	692.0 ± 12.0c	21.2 ± 1.2b	666.5 ± 13.5^c^	220.0 ± 10.0^a^	247.5 ± 12.5^a^
	Matrouh	30.0 ± 0.6^a^	611.0 ± 17.3b	19.7 ± 1.1b	592.6 ± 9.6^b^	279.3 ± 14.6^b^	299.0 ± 11.2^b^
	Mamourah	N/A	N/A	N/A	N/A	N/A	N/A
	Leghorn	32.0 ± 0.6^a^	505.2 ± 7.1a	14.8 ± 0.9a	496.2 ± 15.1a	207.0 ± 11. 8^a^	257.0 ± 9.2^a^
P3	Fayoumi	32.0 ± 1.0^a^	725.8 ± 12.5^c^	21.8 ± 0.9^c^	484.2 ± 12.5^c^	233.0 ± 11.4^a^	262.5 ± 8.1^b^
	Matrouh	30.5 ± 0.5^a^	494.0 ± 4.0^a^	15.2 ± 0.3^ab^	477.8 ± 5.1^a^	234.3 ± 4.0^a^	217.5 ± 1.4^a^
	Mamourah	38.2 ± 0.3^b^	554.2.2 ± 12.4^b^	13.5 ± 0.3^a^	531.9 ± 9.6^b^	244.2 ± 8.5^a^	260.0 ± 6.6^b^
	Leghorn	30.0 ± 2.0^a^	500 ± 10.0^a^	15.7.57 ± 0.6^b^	480 ± 10.0^ab^	215.0 ± 5.2^a^	240.0 ± 5.0^ab^

*The data had been expressed as means ± SE. The values with different letters (a, b, c) within a column represent significant statistical differences P < 0.05. IW: Initial body weight, at one day age. FW: Final body weight at 60 days age. GR: Growth rate. CW: carcass weights. BrW: Breast weight. TW: thigh weight

### Genetic polymorphisms in CAPN1 gene

PCR-SSCP results (Fig. 2C; Table 2) unveiled two distinct patterns, P1 and P2. These patterns were observed in Matrouh, Mamourah, and Leghorn chickens, with the exception of Fayoumi breed that exclusively had P1, while P2 was absent. The frequencies of P1 and P2 in Fayoumi, Matrouh, Mamourah, and Leghorn breeds were recorded as (1.0 and 0.0), (0.8 and 0.2), (0.9 and 0.1), and (0.7 and 0.3), respectively (Table 2).

### Association between the two patterns (P1 and P2) of CAPN1 gene and production traits in four chicken breeds

The influence of patterns P1 and P2 on economic traits, including live body weight, growth rate, carcass weight, breast weight, and thigh weight, was examined (Table 5). Regarding P1, Fayoumi birds carrying this pattern exhibited enhancements in economic traits except for breast weight when compared to the other three breeds. Conversely, Mamourah chickens with P1 displayed the highest breast weight values among the four breeds. As for P2, Matrouh breed with this pattern exhibited significant improvements in various economic traits compared to the other three chicken breeds.

**Table 5:** Effect of SSCP patters of CAPN1 gene on some economic production traits in four chicken breeds

Pattern (P)	Economic traits						
	Breeds	IW	FW	GR	CW	BrW	TW
P1	Fayoumi	31.9 ± 0.7^b^	706.6 ± 19.9^c^	21.6 ± 0.9^c^	677.7 ± 18.2^b^	245.9 ± 10.9^a^	275.2 ± 9.6^a^
	Matrouh	30.1 ± 0.4^a^	536.3 ± 35.5^ab^	16.9 ± 1.3^b^	519.4 ± 16.8^a^	250.3 ± 14.6^a^	247.6 ± 12.1^a^
	Mamourah	38.3 ± 0.3^c^	561.6 ± 14.6^b^	13.7 ± 0.5^a^	539.3 ± 15.1^a^	260.2 ± 13.7^a^	263.3 ± 6.4^a^
	Leghorn	31.6 ± 0.6^b^	512.0 ± 15.4^a^	15.2 ± 0.5^ab^	495.6 ± 14.3^a^	217.9 ± 15.9^a^	260.7 ± 9.6^a^
P2	Fayoumi	N/A	N/A	N/A	N/A	N/A	N/A
	Matrouh	30.5 ± 0.7^a^	733.2 ± 8.8^b^	23.1 ± 0.4^c^	712.5 ± 8.8^b^	316.1 ± 6.9^b^	338.5 ± 8.3^b^
	Mamourah	37.1 ± 1.2^b^	488.1 ± 5.8^a^	12.2 ± 0.7^a^	465.1 ± 2.9^a^	200.2 ± 11.6^a^	230.0 ± 8.7^a^
	Leghorn	31.3 ± 0.7^a^	481.1 ± 13.9^a^	14.3 ± 0.4^b^	458.6 ± 4.5^a^	180.6 ± 9.7^a^	213.7 ± 4.1^a^

*The data had been expressed as means ± SE. The values with different letters (a, b, c) within a column represent significant statistical differences P < 0.05. IW: Initial body weight, at one day age. FW: Final body weight at 60 days age. GR: Growth rate. CW: carcass weights. BrW: Breast weight. TW: thigh weight

### Gene expression at 60 days of age

**Fig. 3:**
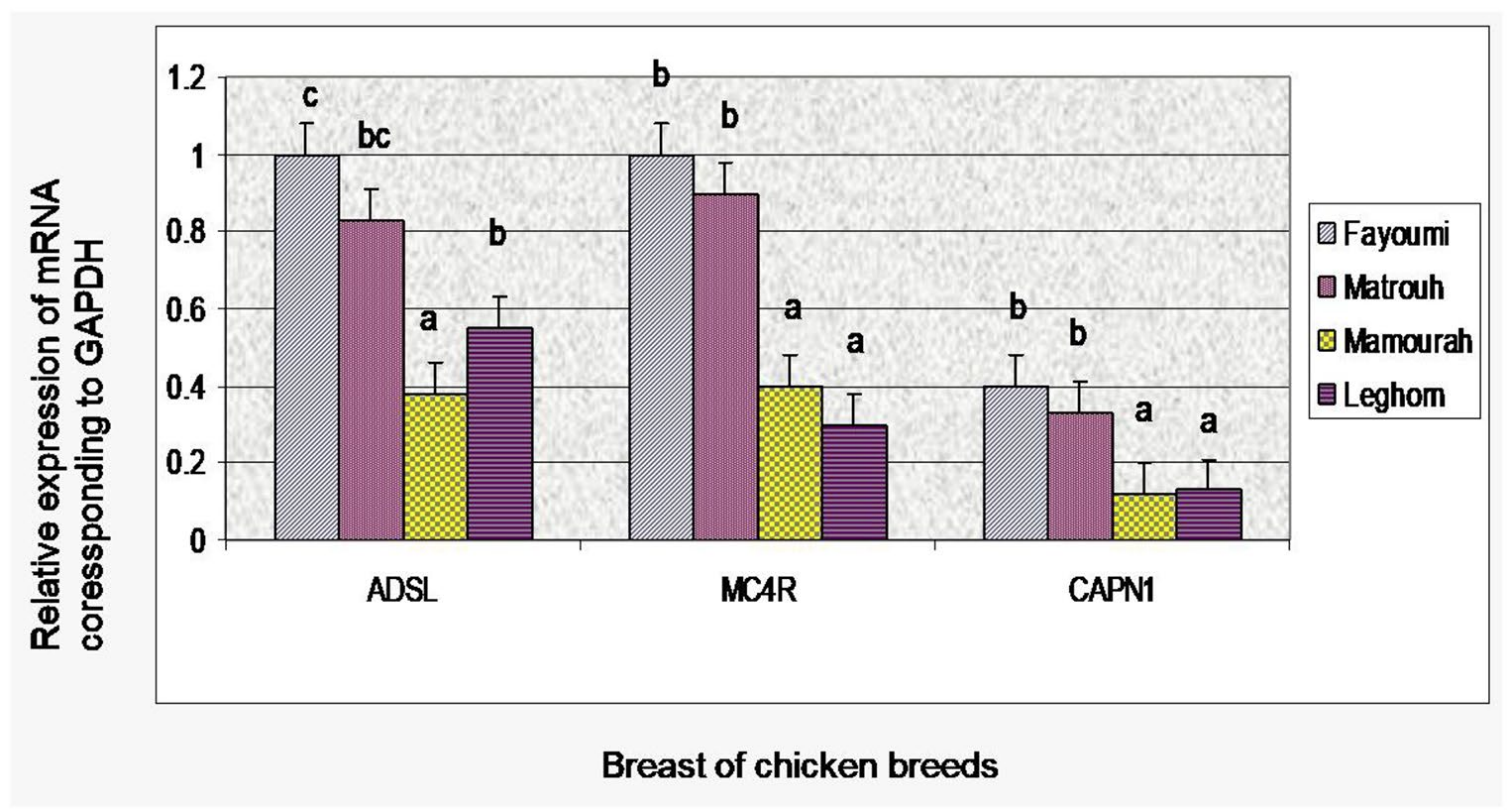
mRNA expression levels of three genes in breast fillet muscle of four chicken breeds (Fayoumi, Matrouh, Mamourah, and Leghorn) at 60 days old

**Fig. 4:**
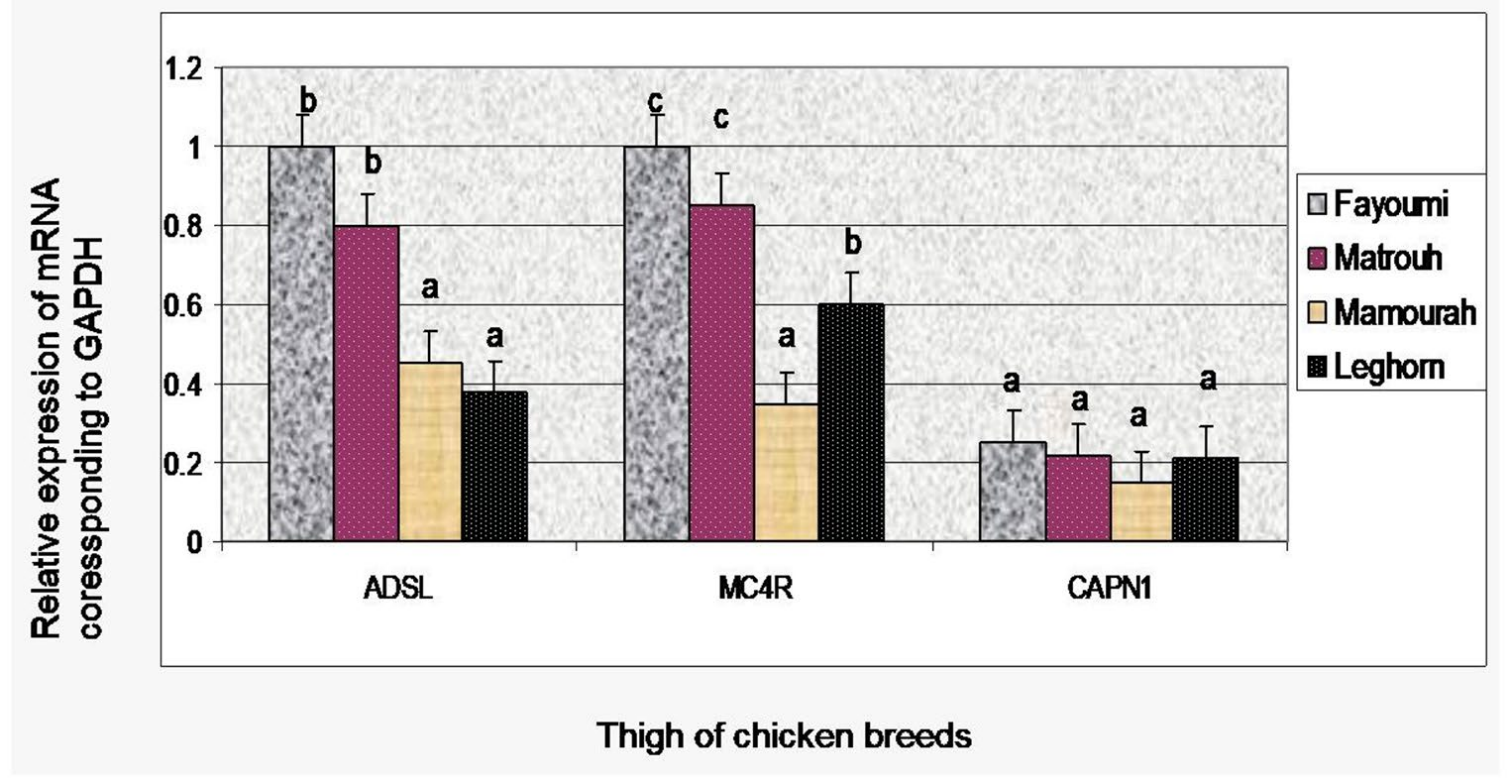
mRNA expression levels of three genes in thigh muscle of four chicken breeds (Fayoumi, Matrouh, Mamourah, and Leghorn) at 60 days old

The expression levels of ADSL, MC4R, and CAPN1 genes in the breast and thigh tissues of the four chicken breeds are depicted in Figs. 3 and 4.

### Gene expression in breast tissues

The relative levels of ADSL and MC4R mRNA expressions were significantly up-regulated in Fayoumi and Matrouh breeds compared to the other two breeds. Additionally, Leghorn breed exhibited a significantly elevated ADSL gene expression compared to Mamourah chickens that displayed the lowest level of expression, whereas gene expression levels of MC4R gene were down-regulated in Mamourah and Leghorn chickens, as Leghorn breed showed the lowest level of MC4R gene expression. CAPN1 mRNA expression exhibited the lowest expressions compared to ADSL and MC4R genes, despite Fayoumi and Matrouh breeds showed a significant increase in its expression, while the lowest level of CAPN1 gene expression was found in Mamourah and Leghorn birds.

### Gene expression in thigh tissues

The ADSL and MC4R gene expressions displayed significant up-regulation in Fayoumi and Matrouh breeds than Leghorn and Mamourah breeds. Conversely, the Leghorn breed exhibited the lowest level of ADSL mRNA expression, in contrast to MC4R gene, Leghorn breed displayed a significantly higher expression relative to Mamourah chickens that recorded the lowest expression. The expression of the CAPN1 gene was down-regulated. However, no significant differences were observed between the four breeds (Fayoumi, Matrouh, Mamourah, and Leghorn). The lowest relative level of CAPN1 mRNA expression was observed in Mamourah breed.

## Discussion

### Polymorphism analyses

In the present work, our comprehensive investigation embraced genetic polymorphism patterns of ADSL, MC4R, and CAPN1 genes in four distinct chicken breeds using PCR-SSCP. Different patterns have been identified in the three genes, each representing different genotypes or alleles [2, 13, 14]. Based on our findings, the ADSL gene exhibited the highest level of genetic diversity, followed by MC4R and CAPN1. Genetic polymorphisms within the ADSL gene revealed five patterns. Pattern 1 (P1) was prevalent in the majority of Fayoumi, P2 in Matrouh, and P3 in Leghorn. Interestingly, these findings align with previous studies that detected genetic polymorphisms using PCR-SSCP. For instance, Kubota et al. [2] and Zhang et al. [25] detected different genotypes (GG, GT, and TT genotypes) and (CC, CT, and TT genotypes in exon 2), respectively within the ADSL gene. These patterns were associated with different levels of BW, BrW, TW, and levels of inosine 5́-monophosphate (IMP) in chickens. The elevated IMP component was associated with a positive effect on enhancing chicken body weight [2, 25, 28]. Consequently, genetic polymorphism within the MC4R gene presented three distinct patterns, with P3 prevailing among the majority of Fayoumi breed chickens. Our findings are in accordance with results of several studies on chickens and farm animals. Li and Li [13] determined Genetic polymorphisms in MC4R gene (AA, AB and BB genotypes) in Arbor Acres chickens, and observed that the birds with BB genotype were discriminated with significant increase in BW and CW compared to birds with the other genotypes. Also, Kubota et al. [2] confirmed genetic polymorphisms (AC and CC genotypes) in MC4R gene in Korat chickens. Birds that carried AC genotype demonstrated significantly heavier BW than birds with CC genotype. Qiu et al. and Wang et al. [20, 21]observed a significant association between polymorphisms in MC4R gene and carcass traits in different chicken breeds. This was shown to be due to the effect of MC4R polymorphisms on the protein activities and functions related to body weight in chickens [2, 13]. Moreover, in farm animals including pigs and bovine, significant correlations between the polymorphisms in MC4R gene and growth traits such as: growth rate, back fat, and feed intake were reported [14, 32–35]. Also, in cattle, Huang et al. [36] detected five polymorphisms in MC4R gene at positions 19 (C/A), 20 (A/T). 83 (T/C), 128 (G/A), and 1069 (G/C). The last one (G/C) was found to be significantly associated with back fat thickness as compared to the other four polymorphisms. In contrast, the CAPN1 gene displayed the lowest level of genetic diversity across the four chicken breeds, manifesting only two patterns (P1 and P2). Regarding CAPN1 gene, our findings were supported by results of previous studies on different chicken breeds using either PCR-SSCP or sequence analysis. Felicio et al. [15] observed CAPN1 polymorphisms in different broiler breeds, including TA, CT, and TC genotypes. TC genotype was correlated with increased body weight including the weight of carcass, breast, and thigh as compared to the other genotypes. Also, in Da-Heng high-quality broiler, Zhou et al. [17] revealed genetic polymorphisms in CAPN1 including AA, AG, GG genotypes. Their results demonstrated that chickens with GG genotype possessed greater live weight, carcass weight, breast muscle weight, leg muscle weight, and abdominal fat weight comparing to those carrying the two other genotypes. Moreover, Our results are in coincidence with those reported by Kubota et al. [2] who showed polymorphisms (AA, AB, and BB genotypes) in CAPN1 gene of Korat chickens, with birds carrying BB genotype had a significantly (P < 0.05) heavier body weight in comparison with those carrying AA or AB genotypes.

### Gene expression analyses

Our exploration extended beyond polymorphism analysis to gene expression levels of the three genes in breast and thigh tissues at 60 days of age, a pivotal stage for market weight. This provides deeper insights into the relation between these candidate genes and chicken economic traits such as FW, GR, CW, BrW, and TW. This approach aligns with the research strategy employed by Liu et al. [37] who investigated the expression of five candidate genes (TPD52L1, FABP7, NCOA7, ASF1A, and GJA1) and found a positive correlation between GJA1 gene expression and breast muscle weight. The present results showed different expression levels of all studied genes (ADSL, MC4R. and CAPN1) in the four chicken breeds. In breast tissues, ADSL and MC4R genes exhibited significant up-regulation in Fayoumi and Matrouh breeds, potentially contributing to their robust growth and weight gain, whereas MC4R gene indicating a breed-specific role in growth regulation. Conversely, CAPN1 gene expression was lower in most breeds, while Fayoumi manifested the highest expression level. This trend resonates with Piorkowska et al. [38], who reported higher CAPN1 mRNA levels in the early age of fast-growing chickens with softer breast muscles. Thigh tissues also revealed compelling insights into gene expression patterns. Fayoumi and Matrouh breeds also exhibited overexpression of ADSL, potentially improving their metabolic pathways and energy utilization, as indicated by their higher average body weights. The Fayoumi breed again stood out with high significant expression of the MC4R gene, further affirming its role in facilitating growth. In contrast, CAPN1 gene expression was down-regulated in most breeds, with a slight elevation in Fayoumi. Interestingly, the polymorphism analysis revealed considerable diversity among the breeds in terms of gene variants, especially for the ADSL gene, which exhibited various polymorphic patterns in different breeds. This extensive diversity suggests the potential for breed-specific adaptation and selective pressures, contributing to the observed variations in growth and body composition traits.

This aligns with the gene expression results, where Fayoumi and Matrouh, with their heightened gene expression levels, showcase superior growth traits compared to the other two breeds. Our findings harmoniously resonate with Kubota et al. [2], who also correlated gene expressions of MC4R, CAPN1, and ADSL with genetic polymorphisms and body weight in Korat chickens across various developmental stages. The MC4R gene plays a pivotal role in regulating appetite and energy expenditure across various species. The elevated expression of MC4R in the Fayoumi breed, followed by Matrouh, suggests an enhanced regulatory mechanism in these breeds, promoting efficient energy utilization and appetite control. This likely contributes to their superior growth and body composition traits, in alignment with their comparatively higher average body weights. Moreover, MC4R expression pattern is consistent with its polymorphism profile that exhibited three distinct genetic patterns, with P3 being prevalent among the majority of Fayoumi breed chickens.

The CAPN1 gene, which displayed the lowest genetic diversity across the four chicken breeds, correspondingly exhibited the lowest gene expression within the breast and thigh muscles. This trend persisted despite the relatively higher GR, BrW, and TW. The higher expression of CAPN1 in Fayoumi and Matrouh suggests that these breeds possess a robust regulatory mechanism for muscle development, aligning with their superior growth and body composition characteristics. These observations can potentially be attributed to the intrinsic role of the CAPN1 gene in myofibrillar protein and myosin degradation [23]. Furthermore, the gene’s documented association with body weight in avian species adds weight to these findings [2, 17, 23]. Nevertheless, the overall low expression of the CAPN1 gene can be explained by previous studies that reported higher CAPN1 expression during the early growth stages [39]. This phenomenon could contribute to the observed decline in gene expression without exerting a significant impact on body weight, potentially facilitating rapid growth during the initial stages before the expression decreases.

## Conclusion

Our study unveils the intricate relationship between genetic polymorphism, gene expression, and economically important traits within poultry breeds. Levels of gene expression, and the unique patterns of the ADSL, MC4R, and CAPN1 genes, highlight their roles in influencing and shaping specific phenotypic traits. This comprehensive understanding enriches our comprehension of the genetic factors driving diversity across poultry populations and offers insights for developing targeted breeding strategies to enhance desirable traits.

## Data Availability

The data and materials described in the manuscript, including all relevant raw data, will be freely available to any scientist wishing to use them for non-commercial purposes, without breaching.
